# Fresh Air: Dyspnœa

**Published:** 1888-01

**Authors:** 


### FRESH AIR: DYSPNŒA.

APIS.—Dyspnœa; seems as if patient cannot survive for want of air; has to be fanned to be kept alive.

CARBO VEG.—Patient desires to be fanned; wants air; with respiratory troubles, and also after loss of fluids.

CHINA.—Desires to be fanned and to have fresh air; in labor and after loss of fluids.

CISTUS.—In evening, soon after lying down, a sensation as if ants were running through the whole body; then anxious, difficult breathing; is obliged to get up and open window; fresh air relieves him; immediately on lying down again these sensations return.

PLUMBUM.—Sits up at night and opens the window.

PULSATILLA.—Wants doors and windows open; in labor and in other troubles.

SULPHUR.—Wants doors and windows open; chiefly in respiratory troubles.

